# Antidiabetic Effect of *Phanera strychnifolia* (Craib) K. W. Jiang, S. R. Gu, & T. Y. Tu Extracts in Streptozotocin‐Induced Diabetic Rats by Upregulating Insulin Secretion and Glucose Transporter 2 and 4 Protein Expression

**DOI:** 10.1155/sci5/8867739

**Published:** 2026-02-26

**Authors:** Kunwadee Lao-On, Udom Lao-On, Anunya Suksanga, Rungruedee Kimseng, Rahni Hossain, Kingkan Bunluepuech

**Affiliations:** ^1^ Department of Medical Technology, Faculty of Allied Health Sciences, Thaksin University, Phatthalung, 93210, Thailand, tsu.ac.th; ^2^ School of Allied Health Sciences, Walailak University, Nakhon Si Thammarat, 80160, Thailand, wu.ac.th; ^3^ Research Excellence Center for Innovation and Health Product, Walailak University, Nakhon Si Thammarat, 80160, Thailand, wu.ac.th; ^4^ Departments of Pharmacology and Nutritional Sciences, Sanders-Brown Center on Aging, University of Kentucky, Lexington, Kentucky, USA, uky.edu

**Keywords:** antidiabetic, GLUT2, GLUT4, insulin, *P. strychnifolia*

## Abstract

Diabetes mellitus, a condition characterized by hyperglycemia, poses significant global health concern. Despite the availability of several antidiabetic drugs, the search for new therapeutic agents with fewer side effects and better efficacy continues. *Phanera strychnifolia* (Craib) K. W. Jiang, S. R. Gu, & T. Y. Tu, a medicinal plant traditionally used in Southeast Asia, has gained attention for its bioactive components. Two major compounds isolated, namely, 3,5,7‐trihydroxychromone‐3‐O‐α‐L‐rhamnopyranoside and 3,5,7,3′,5′‐pentahydroxy‐flavanonol‐3‐O‐α‐L‐rhamnopyranoside, have shown promise as antihyperglycemic agents in human intestinal epithelial Caco‐2 cells. It had been demonstrating antidiabetic and antioxidant activities of its aqueous extract in both in vivo and in vitro studies with limited information regarding their antihyperglycemic effect on insulin secretion and glucose transporter expression. Therefore, this study aims to evaluate the impact of *P. strychnifolia* extracts on blood glucose levels in diabetic rats and to investigate the expression of glucose transporter proteins (GLUT2 and GLUT4) and insulin production in relevant tissues to elucidate the mechanisms of improved glucose uptake and utilization. The antidiabetic effect of *P. strychnifolia* was determined by histological staining and immunocytochemical localization of insulin, GLUT2, and GLUT4 in pancreatic islets and the heart. Additionally, toxicity assessment was conducted over a 63‐day administration by observing biochemical parameters and histological changes. *P. strychnifolia* demonstrates nontoxic characteristics, as evidenced by the absence of mortality and clinical toxicity signs at the 400 mg/kg dose after 63 days of treatment. In diabetic rats, administration of 100 and 200 mg/kg of *P. strychnifolia* for 14 days significantly reduced blood glucose levels by approximately 45.65% and 41.01%, respectively, compared to the diabetic control group. Both doses effectively reduced lipid droplets in the liver, indicating decreased tissue injury. *P. strychnifolia* demonstrates significant antihyperglycemic activity and beneficial effects on insulin and glucose transporter protein expression in diabetic rats, with no observed toxicity. These findings suggest its potential as a therapeutic agent for diabetes management.

## 1. Introduction

Diabetes mellitus (DM) is a widespread and escalating global health challenge, presenting significant problems in both developed and developing nations. Type 2 DM is characterized by insulin resistance and beta‐cell dysfunction, leading to elevated blood sugar levels [1]. It is associated with complications such as cardiovascular problems, nephropathy, neuropathy, and vision impairments. The worldwide prevalence of diabetes is increasing, with projections indicating a rise to 529 million by 2023 and 1.3 billion by 2050 [2].

Diabetes management focuses on blood glucose control through lifestyle changes, diet, physical activity, and sometimes oral medications for type 2 diabetes. Traditional Thai medicinal recipes are gaining attention as an alternative to modern medicines due to their potential to reduce side effects such as hypoglycemia, weight gain, gastrointestinal disturbances, lactic acidosis, liver problems, heart issues, and chronic kidney disease (CKD). The family of facilitative glucose transporters (GLUTs) plays a crucial role in governing the uptake and metabolism of glucose for the regulation of blood glucose levels [3]. When blood glucose levels rise, pancreatic beta cells detect this increase through a process reliant on GLUT2 and respond by boosting insulin secretion [4]. Consequently, insulin attaches to its receptors, enhancing glucose transport into skeletal muscle, adipose tissue, and the heart. This process is primarily facilitated by the acute relocation of GLUT4 transporter vesicles to the cell membrane and the inhibition of hepatic gluconeogenesis. These combined regulatory pathways lead to the removal of glucose from the blood circulation [5]. In type 2 diabetes, the hallmark trait is insulin resistance, where tissues throughout the body display reduced sensitivity to higher insulin levels in the blood. Consequently, this condition results in persistently elevated blood glucose levels, known as hyperglycemia [6].

*P. strychnifolia*, a member of the Fabaceae family, is commonly found throughout Thailand, especially in dry deciduous forests [7]. It is rich in polyphenols and flavonoids and is traditionally used for its diverse pharmacological properties, including antidiabetic, anti‐inflammatory, anticancer, and detoxifying effects [8]. Traditionally, *P. strychnifolia* stems, roots, and leaves were used in food, healthy drink products, dietary supplements, and Thai traditional medicine for immunization, diet, flu relief, reducing alcohol issues, and treating fever [9]. The study by Noonong et al. reported that key compounds such as 3,5,7‐trihydroxychromone‐3‐O‐α‐L‐rhamnopyranoside and 3,5,7,3′,5′‐pentahydroxy‐flavanonol‐3‐O‐α‐L‐rhamnopyranoside derived from *P. strychnifolia* have the potential to serve as antidiabetic agents. They achieve this by inhibiting α‐glucosidase, reducing glucose absorption in the enterocytes of the small intestine in Caco‐2 cells as a model of human intestinal epithelial cells. This glucose absorption reduction is accomplished by suppressing gene expression related to glucose transporters and inhibiting binding sites for SGLT1 and GLUT2 [10]. Another study indicates that *P. strychnifolia* leaf extract significantly enhances glucose uptake, improves glucose tolerance, elevates insulin levels, increases GLUT4 expression, and exhibits antioxidant properties, indicating its potential as an antihyperglycemic agent [11]. Furthermore, evidence suggests that compounds isolated from *P. strychnifolia* stem and leaves, such as astilbin, trilobatin, yanangdaengin, resveratrol, epicatechin, quercetin, and gallic acid, have shown potential antidiabetic effects by inhibiting enzymes and promoting glucose storage [12, 13]. The antioxidant capacity of *P. strychnifolia* extracts may also contribute to its antidiabetic mechanisms [11].

However, to date, there is limited information on their impact on insulin secretion, glucose transporter expression, or systemic glucose regulation in diabetic models. This gap in knowledge justifies the need for a comprehensive study of P. *strychnifolia* in an animal model. Therefore, this research aims to evaluate the impact of P. *strychnifolia* on diabetic rats induced by a high‐fat diet (HFD) and a low dose of streptozotocin (STZ). The assessment includes measuring its antidiabetic effects and using immunohistochemistry to evaluate insulin and GLUT2 and GLUT4 expressions with two doses of P. *strychnifolia* stem extract (100 mg/kg and 200 mg/kg) compared to metformin, a standard diabetic medication. Additionally, the study concludes with an evaluation of the toxicity of P. *strychnifolia* in Wistar rats.

## 2. Materials and Methods

### 2.1. Plant Materials

The stems of *P. strychnifolia* were gathered in 2020 from the Suan Ya Tai Tongnoppakhun herbal garden located in Chonburi Province. A voucher specimen (SKP 072021901) is presently archived at the Herbarium of the Department of Pharmacognosy and Pharmaceutical Botany within the Faculty of Pharmaceutical Sciences at Prince of Songkla University in Thailand. *P. strychnifolia* was identified by Assoc. Prof. Dr. Oratai Neamsuvan taxonomy, Faculty of Traditional Thai Medicine, Prince of Songkla University, who confirmed the corrected name based on https://powo.science.kew.org/taxon/urn:lsid:ipni.org:names:77313975-1. Dr. Kingkan Bunluepuech had permission for collected *P. strychnifolia* for this research.

### 2.2. *P. strychnifolia* Extracts Preparation

*P. strychnifolia* (3.5 kg) was subjected to water extraction (40 L), and the resulting solution was evaporated using a freeze dryer. This process yielded 1080 g of crude extract, the weight of which was measured to calculate the 30% yield in the rat model study.

### 2.3. Toxicity Study of the Crude *P. strychnifolia* Extract

Previous research by Somsak et al. reported oral administration of *P. strychnifolia* (or *B. strychnifolia*) leaf extract at doses up to 3000 mg/kg caused no signs of toxicity in mice, whereas behavioral alterations were observed at a dose of 6000 mg/kg [14]. Additionally, Peerarat et al. demonstrated that oral administration of *P. strychnifolia* stem ethanol extract at doses ranging from 300 to 5000 mg/kg caused no observable toxic effects. Consequently, the LD_50_ value of the extract in mice was estimated to be greater than 5000 mg/kg [15]. Based on this, the middle dose of 200 mg/kg (approximately 1/25th of the LD_50_) was considered safe and pharmacologically significant. The low dose (100 mg/kg) was chosen as half of the middle dose. The toxicity test was performed by administering the extract 400 mg/kg (twice of the middle dose) orally throughout the experimental period (63 consecutive days), which corresponded to the total duration of diabetes induction (HFD feeding and STZ injection) and treatment in this study, to ensure that the extract caused no‐observed‐adverse‐effect level (NOAEL). Following the administration of the extract, each animal was closely monitored for signs of toxicity, including changes in behavior, body weight, and food and water consumption. Additionally, biochemical and histopathological analyses were conducted to detect any adverse effects on organ function and structure. This method ensures a comprehensive assessment of the extract’s potential toxicological impact over a prolonged exposure period.

### 2.4. Animal Handling

Male Wistar rats, 6 weeks old, weighing 200–300 g, were supplied by Nomura Siam International Channel Limited Company. The animal shelter was raised at Walailak University, Thailand, at constant temperature (22°C–25°C), with a relative humidity of 50%–60% and a 12 h light–dark cycle. Rats were acclimatized for 7 days before the experiment. They were housed in ventilated cages and fed diet and water *ad libitum*. The extract was dissolved in 0.9% sodium chloride and was administered to the animals using the oral gavage method. All rats were euthanized at the end of the experiment using an intraperitoneal injection of sodium pentobarbital (120–150 mg/kg), in accordance with the euthanasia guidelines of the AVMA (American Veterinary Medical Association) and CCAC (Canadian Council on Animal Care). The animal care and experimental protocol were approved by the Walailak University Institutional Animal Care and Use Committee (WU‐IACUC‐65035), Walailak University.

### 2.5. Animal Experimental Design

According to the animal NOAEL of *P. strychnifolia* extract and the standard drug metformin was administered at a dose of 250 mg/kg body weight [16]. Therefore, the chosen doses of *P. strychnifolia* extract (100 and 200 mg/kg body weight) were investigated to be comparable to, although slightly lower than, the effective dose of the standard drug to assess its therapeutic potential under safe and relevant conditions. The required sample size was estimated using G^∗^ Power version 3.0.10 (effect size (*f*) = 0.85, *α* = 0.05, power (1–*β*) = 0.9, and number of groups = 6). The rats were randomly divided into six groups (*n* = 10 per group) as follows:

 Group I: normal control administered 0.9% sodium chloride (NaCl)
 Group II: normal rats administered *P. strychnifolia* (400 mg/kg)
 Group III: STZ‐induced diabetic control administered 0.9% NaCl
 Group IV: diabetic rats administered the standard drug metformin (250 mg/kg)
 Group V: diabetic rats administered *P. strychnifolia* (100 mg/kg)
 Group VI: diabetic rats administered *P. strychnifolia* (200 mg/kg)

### 2.6. Induction of Diabetes Type 2

The induction of diabetes was performed by providing a HFD for 21 days consisting of 55% of energy from lipids derived from pork lard and soybean oil, 31% from carbohydrates, and 14% from protein (5.2 kcal/g) [16]. On day 22, HFDs were administered a single intraperitoneal injection of STZ (40 mg/kg), while the control rats received the vehicle alone. Fasting blood glucose (FBG) was measured 7 and 14 days after STZ injection. Rats with FBG levels greater than 270 mg per deciliter (mg/dL) were considered diabetic and used for the experiment [17, 18].

### 2.7. Fasting Blood Glucose (FBG) Analysis

FBG levels were estimated by the enzymatic glucose oxidase method using a commercial glucometer (Accu‐Chek Performa, Roche Diabetes Care, Switzerland). Blood was drawn from the rats at the tail end to measure blood glucose. Rats received designated agents via oral gavage every day for 14 days, and FBG levels were assessed on days 7 and 14 to determine antihyperglycemic activity.

### 2.8. Biochemical Analysis

Biochemical parameters were observed, and the levels of serum parameters, including serum urea, uric acid, serum creatinine, cholesterol, triglycerides, HDL, LDL, aspartate aminotransferase (AST), alanine aminotransferase (ALT), and alkaline phosphatase (ALP), were elevated or declined when compared with those in the normal group [19]. A TBA‐120FR automatic chemical analyzer analyzed the biochemical parameters according to the manufacturer’s instructions [20, 21].

### 2.9. Histopathological Examination

Liver, kidney, and pancreas specimens were collected and fixed in 10% neutral buffered formalin (NBF) for 24 h, dehydrated, and embedded in paraffin wax. The sections were stained with hematoxylin solution and eosin (H&E). Slide observation and imaging were performed at magnifications of 10x and 40x for histological analysis [22]. Histopathology for the liver cells showed that the changed tissue had areas of necrosis, vacuolization of hepatocytes, fatty changes, parenchymal architecture disruption, deposition of collagen fibers, dilatation of sinusoids, central vein congestion, and inflammatory infiltration when compared with normal cells. Kidney cells were observed to have a necrotic tubule wall, necrosis in the glomerulus and tubules, degeneration, accumulation of hyaline material, and deposition of collagen compared with normal cells [22]. Heart tissues were observed to have hypertrophied and disorganized myocytes and interstitial fibrosis [23]. Pancreatic tissues exhibit hyperplasia, metaplasia, islets of Langerhans, and malignant transformation [24].

### 2.10. Immunohistochemistry

The pancreas and heart specimens were preserved in formaldehyde, embedded in paraffin, and sliced into sections with a thickness of 10 mm. The sections were soaked in a Tris‐buffered saline (TBS) solution mixed with a 3% hydrogen peroxide methanol solution for 30 min to prevent natural peroxidase activity. This was followed by incubation at room temperature for 30 min. The slides were then incubated with polyclonal‐insulin antibody (1:500), GULT2 (1:1000), and GLUT4 (1:1000) overnight at 4°C. Then, the slides were rinsed three times with TBS for 3 min. The slides were exposed to a secondary antibody conjugated with HRP (1:1000) at room temperature for 2 h. Subsequently, they were rinsed with 3,3′‐diaminobenzidine tetrahydrochloride (DAB) in preparation for imaging. The images were acquired using a light microscope with a camera (Olympus BX43, Japan) at 200X and 400X magnification.

To measure image intensity, five randomly selected fields per section were captured at 200× magnification. As the tissues were stained with DAB without counterstaining, the images were analyzed using ImageJ software. The threshold value was then adjusted to remove background signal while remaining the true DAB signal. This threshold was fixed and applied to all images. Mean intensity of DAB‐positive regions was calculated relative to the total tissue area [25].

### 2.11. Ethical Approval

The study was performed after receiving approval from the Walailak University Institutional Animal Care and Use Committee (Ref. No. WU‐ACUC‐65035), Walailak University.

### 2.12. Statistical Analysis

The statistical analysis was performed by comparing the diabetic group with the diabetic groups treated with *P. strychnifolia* extract. One‐way analysis of variance (ANOVA) followed by Tukey’s post hoc test for multiple comparisons was conducted using GraphPad Prism software version 8.4.2. Data are presented as mean ± standard error of the mean (SEM). Statistical significance was set at *p* < 0.05, indicating a 95% confidence level.

## 3. Results

### 3.1. Effect of *P. strychnifolia* on Toxicity in Rats

**FIGURE 1:**
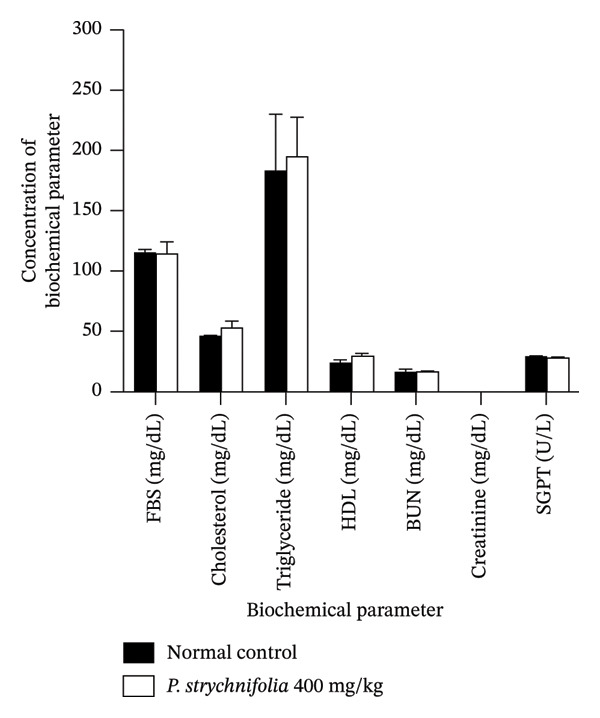
Effect of administration of *P. strychnifolia* 400 mg/kg continuously for 63 days on the biochemical parameters of rats. The results showed no significant toxicological effects on liver and kidney functions.

The nontoxic characteristics of *P. strychnifolia* were demonstrated by the absence of mortality at the 400 mg/kg dose after 63 days of treatment. The administration of *P. strychnifolia* extract at 400 mg/kg did not result in significant weight loss, a common indicator of severe toxicity, and no weight alterations were observed in kidneys. The liver weight in the toxicity group was slightly higher than that in the control group indicating no significant hepatomegaly (Table 1). In addition, there were no observable changes in biochemical parameters, including serum urea, creatinine, cholesterol, triglyceride, HDL, AST, and FBS levels, compared to the control group (Figure 1).

**TABLE 1:** Effect of administration of *P. strychnifolia* 400 mg/kg continuously for 63 days on the body weight and organ weight of rats.

Group	Body weight (g)	Liver weight (g)	Kidney weight (g)
Control group	413.19 ± 60.58	13.07 ± 0.79	2.96 ± 0.15
Toxicity group (400 mg/kg)	466.92 ± 44.44	13.99 ± 1.55	2.76 ± 0.19

**FIGURE 2:**
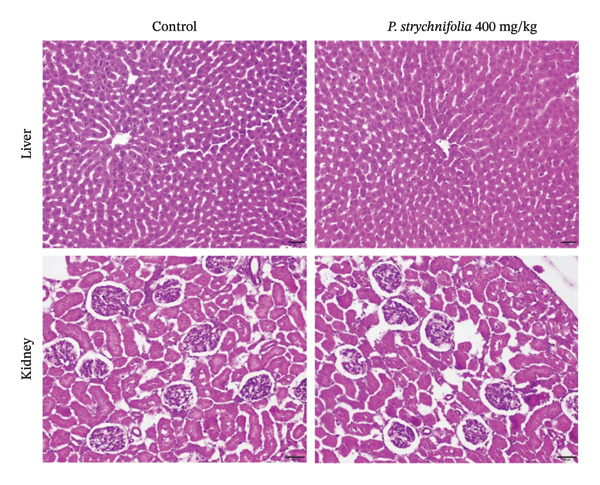
Effect of administration of *P. strychnifolia* 400 mg/kg continuously for 63 days on histopathological changes of the liver and kidney in rats with H&E staining (200X magnification). The results showed no significant toxicological effects on liver and kidney morphology.

A histopathological examination was conducted to assess the impact of the extract on the liver and kidneys (Figure 2). The results demonstrated that the livers of rats treated with 400 mg/kg for 63 days exhibited normal morphology, without hypertrophy of hepatocytes or cellular infiltration, as observed in the control group. For the kidney, rats were treated with 400 mg/kg for 63 days, and the normal control group presented a normal kidney morphology with a normal structure and boundary of the glomerulus and a normal size of renal tubules.

### 3.2. Effect of *P. strychnifolia* on Fasting Blood Glucose (FBG)

**FIGURE 3:**
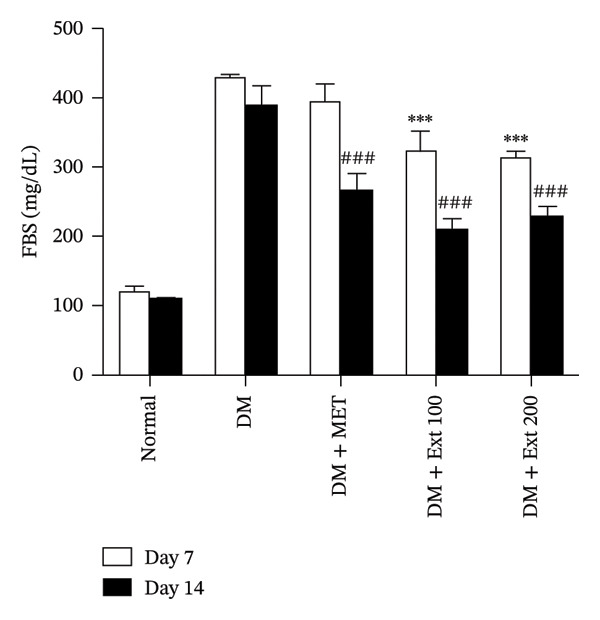
Effect of *P. strychnifolia* (100 and 200 mg/kg) on fasting blood glucose in diabetic rats compared with standard drug metformin (250 mg/kg). (^∗∗∗^ *p* < 0.001 vs. diabetic control group on day 7 and ^###^ *p* < 0.001 vs. diabetic control group on day 14).

The administration of *P. strychnifolia* (100 and 200 mg/kg) significantly reduced FBG levels in diabetic rats on both days 7 and 14, compared to the diabetic control group. However, there was no significant difference between the 100 and 200 mg/kg extract‐treated groups on days 7 and 14. The effect was dose‐dependent, with the higher dose showing more pronounced antihyperglycemic activity. In comparison, the standard drug, metformin (250 mg/kg), also resulted in a significant reduction in FBG levels, though the reduction was more prominent on day 14. Despite these effects, neither *P. strychnifolia* dose nor metformin was able to fully normalize blood glucose levels to those observed in the normal control group (Figure 3).

### 3.3. Effect of *P. strychnifolia* on Histopathology of the Liver and Pancreas

**Figure 4:**
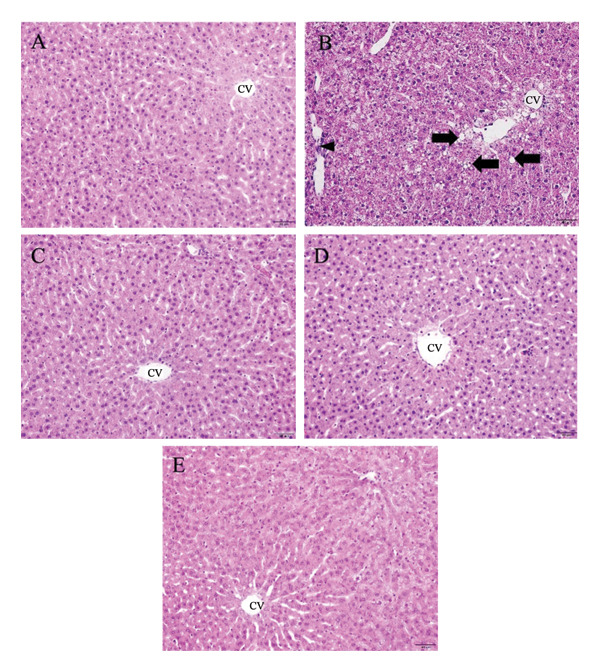
Histopathological appearance of liver cells with H&E staining (200X magnification). (A) Normal control rats had no inflammation or no evidence of liver damage; (B) DM control diabetic rats showed extensive degeneration of hepatocytes, hepatocyte vacuolation (arrow), and inflammatory infiltration (arrowhead); (C) diabetic rats treated with 250 mg/kg of metformin showed normal hepatocytes; (D) diabetic rats treated with 100 mg/kg of *P. strychnifolia* exhibited improved hepatocyte morphology with moderate sinusoidal dilation; (E) diabetic rat group treated with 200 mg/kg of *P. strychnifolia* showed almost the normal appearance of hepatocytes. Scale bars = 50 μm.

The normal histological structure of the liver is observed in Figure 4(A). The liver of diabetic rats showed extensive hepatocyte degeneration, vacuolation, and inflammatory infiltration (Figure 4(B)). Diabetic rats treated with 250 mg/kg of metformin showed nearly normal hepatocyte morphology, with minimal damage (Figure 4(C)). Rats treated with 100 mg/kg of *P. strychnifolia* showed improved hepatocyte structure, though moderate sinusoidal dilation remained (Figure 4(D)). The group treated with 200 mg/kg of *P. strychnifolia* displayed near‐normal liver architecture, with well‐preserved hepatocytes and minimal signs of damage (Figure 4(E)). These results suggest that both metformin and *P. strychnifolia* provide hepatoprotection in diabetic rats, with the higher dose of *P. strychnifolia* showing the most significant recovery.

**FIGURE 5:**
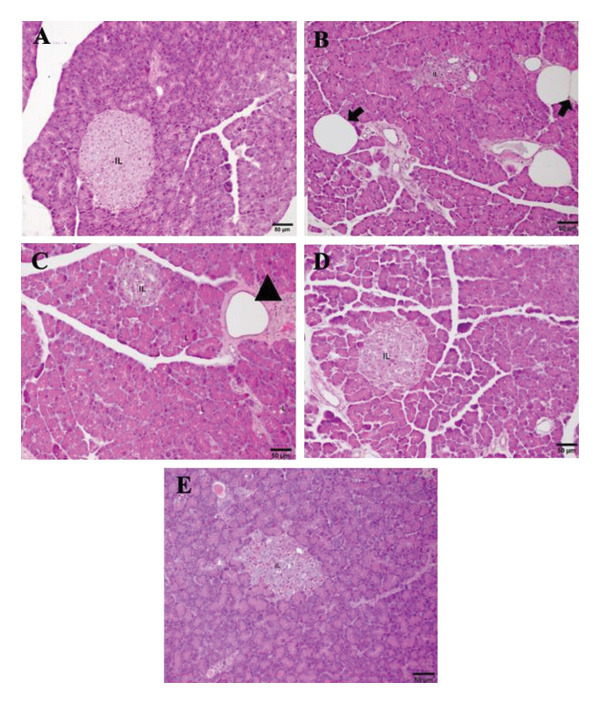
Effect of *P. strychnifolia* extract on histopathology of pancreas in streptozotocin (STZ)‐induced diabetic rats. Histopathological appearance of pancreatic cells with HE staining (200X magnification). (A) Normal control rats displayed typical pancreatic acinar structure and well‐organized islets of Langerhans (IL). (B) The DM control diabetic rat group exhibited reduced islet mass, disorganized pancreatic islets, and lipomatosis (arrow). (C) Diabetic rats treated with 250 mg/kg of metformin showed moderate improvement in islet mass, with blood vessels markedly dilated (arrowhead). (D) Diabetic rats treated with 100 mg/kg of *P. strychnifolia* demonstrated improved morphology of the islets of Langerhans. (E) Diabetic rats treated with 200 mg/kg of *P. strychnifolia* displayed near‐normal appearance of the islets of Langerhans, with a normal cellular composition. Scale bars = 50 μm.

Histopathological analysis of pancreatic tissue revealed notable differences across the experimental groups. In the normal control group, the pancreas exhibited a typical structure with well‐defined pancreatic acini and organized islets of Langerhans, indicative of healthy pancreatic tissue with no signs of damage or disruption (Figure 5(A)). In contrast, the DM group showed shrinkage and disorganization of the islets of Langerhans. Additionally, the presence of lipomatosis was observed, highlighting fatty infiltration into the pancreatic tissue, a common feature in diabetic pathology (Figure 5(B)). Treatment with 250 mg/kg of metformin showed a slight restoration of islet mass. However, blood vessels were markedly dilated (Figure 5(C)). Rats treated with 100 mg/kg of *P. strychnifolia* and 200 mg/kg of *P. strychnifolia* (Figures 5(D) and 5(E)) demonstrated significant improvement in the morphology of the islets of Langerhans, restoring the typical acinar structure and well‐organized islet architecture, with a normal cellular composition, compared to the DM group.

### 3.4. Effect of *P. strychnifolia* on Insulin and GLUT2 and GLUT4 Expressions by Immunohistochemistry

The influence of the number of β‐cells in the pancreas was detected using immunohistochemistry. Staining observations revealed that β‐cells, which produce insulin within the islets of Langerhans were considered as cells with brown‐colored cytoplasm. It was found that rats in the normal group (Figure 6(a)) exhibited normal sizes and staining of the β‐cells in islets of Langerhans and demonstrated insulin expression through immunoreactivity. On the other hand, rats in the DM group (Figure 6(a)) exhibited a noticeable decrease in the size and β‐cell staining in the islets of Langerhans. Diabetic rats treated with metformin and 100 mg/kg and 200 mg/kg of *P. strychnifolia* (Figure 6(a)) showed islet sizes and staining patterns that closely resembled normal rats. When quantifying the intensity using the ImageJ program (Figure 6(b)), it was found that the 200 mg/kg extract significantly increased insulin expression in the pancreas compared to the diabetic group (*p* < 0.0001) and also showed a significantly higher expression than the 100 mg/kg extract‐treated group (*p* < 0.05).

FIGURE 6(a) Immunohistochemical strained section of insulin rat pancreas (200X magnification); (b) quantification of insulin expression intensity in pancreatic islets using ImageJ analysis. Normal was normal control rats; DM control was the diabetic rat group; DM + metformin was the diabetic rat group treated with 250 mg/kg of metformin. DM + Ext. 100 represents the diabetic rat group treated with 100 mg/kg of *P. strychnifolia*. DM + Ext. 200 represents the diabetic rat group treated with 200 mg/kg of *P. strychnifolia*. (^∗^ *p* < 0.05 and ^∗∗∗∗^ *p* < 0.0001 vs. diabetic control group).

**Figure:**
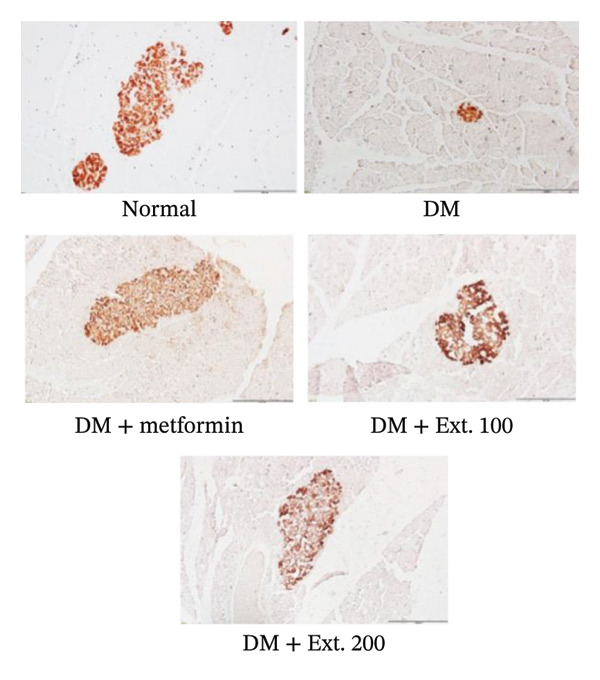
(a)

**Figure:**
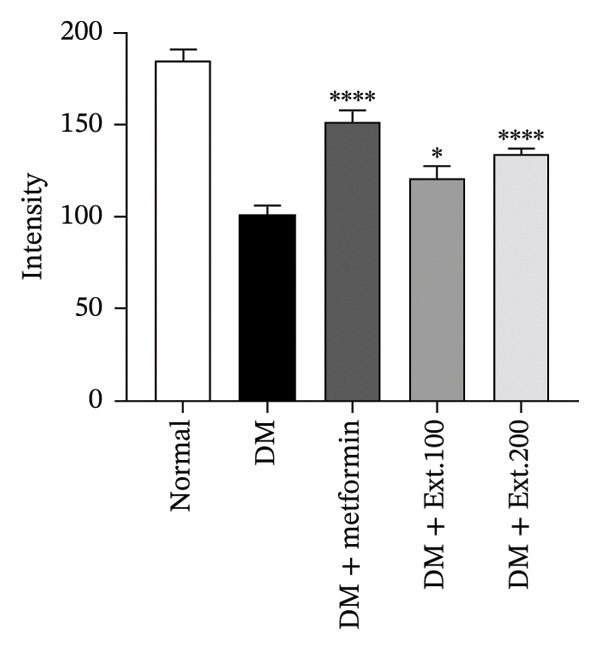
(b)

The evaluation of GLUT2 expression was carried out on the pancreas. The results revealed that rats in the normal group (Figure 7(a)) exhibited normal sizes and staining, along with normal levels of GLUT2 expression within the beta cells in the islets of Langerhans, as indicated by immunoreactivity. Furthermore, diabetic rats treated with metformin and an extract dose of 200 mg/kg (Figure 7(a)) displayed islet sizes and staining patterns that closely resembled those of the normal group. In contrast, diabetic rats in the DM group and the 100 mg/kg group (Figure 7(a)) showed a significantly reduced size of staining. However, when quantifying the intensity using the ImageJ program (Figure 7(b)), it was observed that the 200 mg/kg extract significantly increased GLUT2 expression, and this increase was statistically significant (*p* < 0.0001) when compared to the diabetic rats.

FIGURE 7(a) Immunocytochemical localization of GLUT2 in pancreatic islets (20x magnification). (b) Quantification of GLUT2 expression intensity in pancreatic islets using ImageJ analysis. Normal was normal control rats; DM control was the diabetic rat group; DM + Metformin was the diabetic rat group treated with 250 mg/kg of Metformin. DM + Ext. 100 represents the diabetic rat group treated with of *P. strychnifolia* 100 mg/kg. DM + Ext. 200 represents the diabetic rat group treated with 200 mg/kg of *P. strychnifolia.* (^∗∗∗∗^ *p* < 0.0001 vs diabetic control group, NS = not significant).

**Figure:**
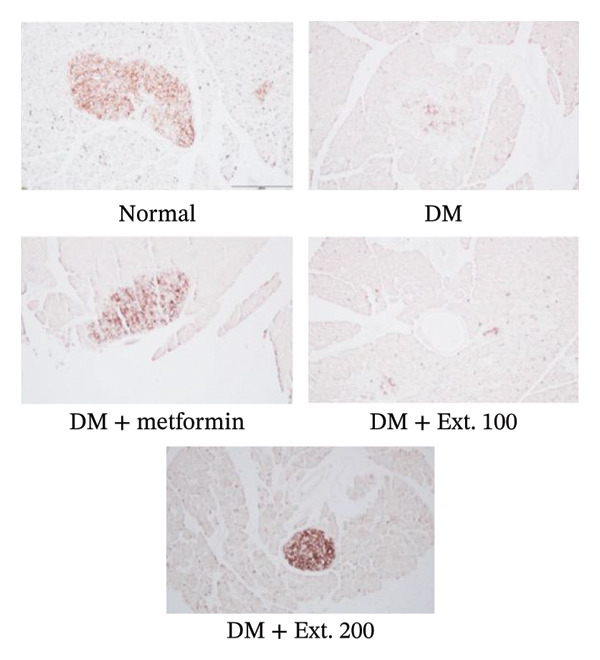
(a)

**Figure:**
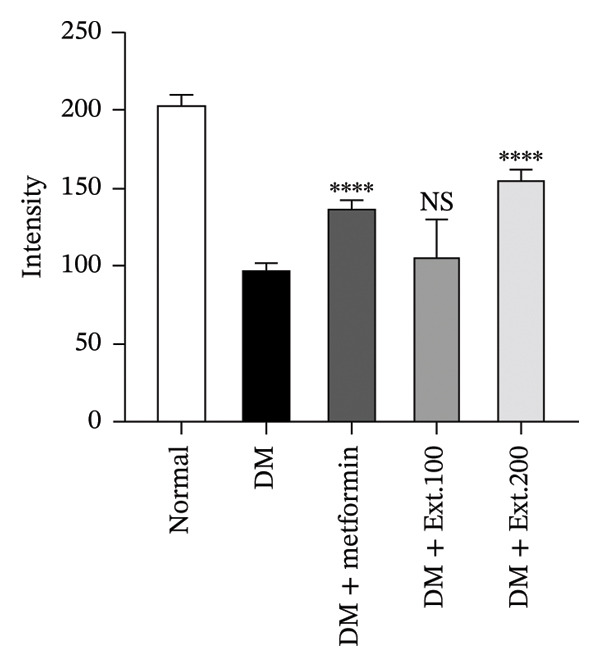
(b)

In addition, the immunohistochemistry study of GLUT4 in cardiac tissue was investigated; normal rats in the control group (Figure 8(a)) displayed normal, strong staining of the cardiac muscle and exhibited GLUT4 expression. In contrast, the diabetic rats in the DM group (Figure 8(a)) had lower staining intensity than those in the control group. However, diabetic rats treated with metformin and extract doses of 100 and 200 mg/kg (Figure 8(a)) showed staining patterns similar to those in the normal group. When quantifying the staining intensity using the ImageJ program (Figure 8(b)), it was evident that the 100 and 200 mg/kg extracts significantly increased GLUT4 expression in the cardiac muscle when compared to the DM group (*p* < 0.0001).

FIGURE 8(a) Immunocytochemical localization of GLUT4 in the heart (200x magnification). (b) Quantification of GLUT4 expression intensity in pancreatic islets using ImageJ analysis. Normal was normal control rats; DM control was the diabetic rat group; DM + metformin was the diabetic rat group treated with 250 mg/kg of metformin. DM + Ext. 100 represents the diabetic rat group treated with 100 mg/kg of *P. strychnifolia.* DM + Ext. 200 represents the diabetic rat group treated with 200 mg/kg of *P. strychnifolia.* (^∗∗∗∗^ *p* < 0.0001 significant vs. diabetic control).

**Figure:**
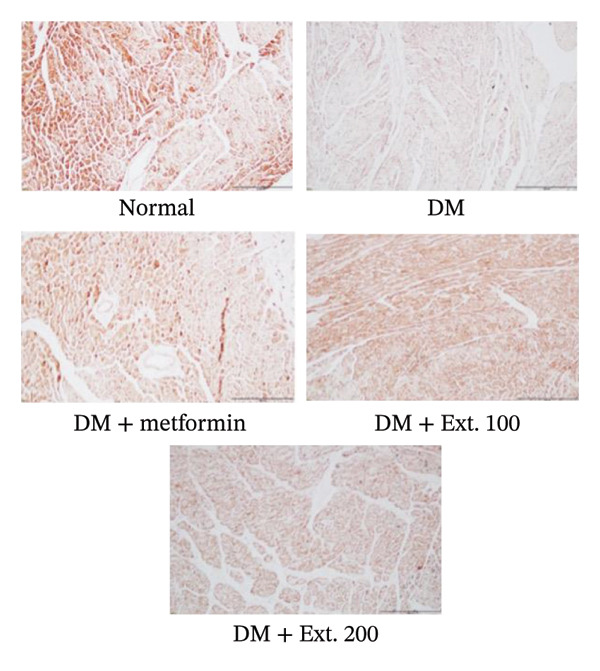
(a)

**Figure:**
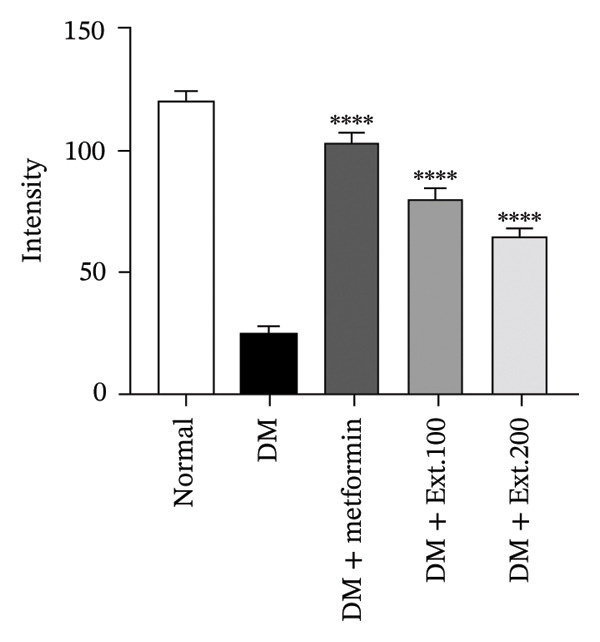
(b)

## 4. Discussion

The toxicity study of 400 mg/kg of *P. strychnifolia* oral administration for 63 days in normal Wistar male rats (Group II) showed the absence of mortality. The body weight and organ weight, including the kidney and liver, were not significantly different from those of the control group (Group I), but the toxicity group showed a slightly increased weight trend more than the normal group. This suggests that administration of *P. strychnifolia* did not affect growth abnormalities, changing internal organ weight or mortality in rats. The biochemical results showed that rats administered *P. strychnifolia* 400 mg/kg for 63 days did not show changes in blood glucose levels, cholesterol, HDL, BUN, creatinine, or SGPT levels compared to the normal control group. The results exhibited a consistent correlation between biochemical values and histopathological findings. Rats administered *P. strychnifolia* displayed normal hepatic morphology, characterized by the absence of hepatocyte hypertrophy, cellular infiltration, fatty change, balloon degeneration, or hepatic necrosis [26]. Specifically, the SGPT level, known to increase when the liver is injured, remained within the normal range in this study [27]. In the examination of the kidneys, no abnormal characteristics were identified, encompassing vascular degeneration, cytoplasmic blebbing, changes in the nucleus indicative of necrosis (pyknosis, karyorrhexis, and karyolysis), interstitial edema, or interstitial inflammation [28]. The BUN level serves as an indicator of kidney function and overall liver health [29]. BUN of rats administered *P. strychnifolia* remained within the normal range. These combined results strongly indicate that *P. strychnifolia* did not induce adverse effects on the liver and kidneys of rats.

Diabetic rats were induced by receiving a HFD for 21 days and a single intraperitoneal injection of STZ (40 mg/kg). After the rats were confirmed to be diabetic, they were treated with 100 and 200 mg/kg of *P. strychnifolia* for 14 days. Following the 14‐day treatment period, both doses of *P. strychnifolia* effectively reduced FBG levels compared to the diabetic control (*p* < 0.05). Moreover, the 100 mg/kg dose of *P. strychnifolia* demonstrated a greater reduction in FBG levels than rats administered metformin (standard medicine) and the 200 mg/kg dose of *P*. *strychnifolia* on day 14. Two key compounds in *P. strychnifolia* associated with diabetes are 3,5,7‐trihydroxychromone‐3‐O‐α‐L‐rhamnopyranoside (compound 1) and 3,5,7,3′,5′‐pentahydroxy‐favanonol‐3‐O‐α‐L‐rhamnopyranoside (compound 2) [10]. These compounds can inhibit α‐amylase and α‐glucosidase, similar to acarbose, a diabetes medication. Additionally, compound 1 demonstrated a superior ability to inhibit α‐glucosidase compared to acarbose, approximately 133% better. This outcome suggests that *P. strychnifolia* helps to reduce the degradation of polysaccharides and disaccharides to monosaccharides (glucose). This is another way to prevent glucose from entering the bloodstream, leading to decreased FBG levels. In addition, both compounds can reduce FBG levels through the SGLT1 and GLUT2 pathways in Caco‐2 cells [10]. Interestingly, although both doses of *P. strychnifolia* extract (100 and 200 mg/kg) significantly reduced FBG levels compared with the diabetic control, there was no significant difference between the two doses. However, the immunohistochemical analysis demonstrated that the 200 mg/kg extract markedly enhanced insulin expression in pancreatic tissues compared to the 100 mg/kg group. This suggests that the higher dose may exert stronger effects at the cellular level by promoting β‐cell function and insulin synthesis.

Diabetes is characterized by metabolic disorders affecting glucose and lipid metabolism, resulting in elevated blood glucose levels [30]. The liver and kidneys, crucial organs in metabolic regulation, play significant roles in this context [31]. The development of nonalcoholic fatty liver disease (NAFLD) is strongly associated with diabetes [32]. Oxidative reactions contribute to the onset of diabetes, and the resulting dysregulated hepatic antioxidant status, along with disturbances in the metabolism of fatty acids and glucose, leads to liver and pancreas damage [33]. STZ has been reported to induce liver toxicity, exhibiting characteristics similar to those observed in NAFLD [34]. Evaluation of NAFLD was conducted by counting the number of fatty droplets combined with considering lipid profiling and liver biochemistry [35]. Droplets in rats treated with 100 and 200 mg/kg of *P. strychnifolia* were significantly lower than those in the diabetic control group. It showed normal architecture compared with that in the normal group. *P. strychnifolia* is mainly composed of polyphenols and flavonoids whose anti‐inflammatory and antioxidant properties are associated with a low prevalence of metabolic diseases, including obesity, hypertension, and insulin resistance [36]. The high content of these compounds may be related to the reduction in fatty droplets, as indicated by the study in a diabetic mouse model [37]. The study showed polyphenols reversed hepatic dysfunction associated with Nesfatin‐1 and glycolipid metabolism. Additionally, another study on a mouse model of a HFD reported that polyphenols can prevent liver fat accumulation by enhancing fatty acid β‐oxidation and reducing lipogenesis through the central role of AMPK regulation as a modulatory protein in lipid metabolism [38].

When rats become diabetic, insulin and GLUT2 expression are reduced [39]. Figures 7 and 8 illustrate the impact of *P. strychnifolia* on insulin and GLUT2 and GLUT4 expressions. The results indicate that both doses of *P. strychnifolia* (100 and 200 mg/kg) significantly increased insulin and GLUT2 and GLUT4 expressions, except at the dose of 100 mg/kg of *P. strychnifolia,* which did not enhance GLUT2 expression. The reason may be that the dosage was not optimal for GLUT2, which corresponds to Salame et al.’s studies, which recorded that chrysin extracted at 40 and 80 mg/kg can increase GLUT2 expression, but 40 mg/kg could enhance GLUT2 lower than a dose of 80 mg/kg [40]. According to Fumagalli et al., gamma‐conglutin extracted from *Lupinus albus* seeds has been shown to reduce blood glucose levels in diabetic rats by enhancing the expression of GLUT2, Pdx‐1 and SLC2A2 genes in pancreatic tissue, bringing them closer to normal levels [41]. On the other hand, the extract from *P. strychnifolia* is rich in polyphenols, which may be the key compound responsible for increasing GLUT4 expression. Romaiyan et al. reported that the extract from olive leaf polyphenols (OLPs) has the potential to increase GLUT4 expression by stimulating Rab8A and Rab13 proteins, which are key regulators for reducing blood glucose and triglycerides and improving insulin function [42].

Several studies have demonstrated that the AMPK and PI3K/Akt pathways, along with insulin receptor substrate‐1 (IRS‐1), play important role in the regulation of GLUT translocation to the plasma membrane and enhancing insulin sensitivity. The AMPK activation is associated with enhancing glucose uptake independently of insulin with GLUT4 translocation from cytosol to the cell membrane resulting in the increasing of muscle glucose uptake [43, 44]. It has been reported that antidiabetic drug metformin increases AMPK phosphorylation and mediates glucose metabolism [45]. Moreover, the IRS‐1/PI3K/Akt signaling system, via its downstream effector PDX‐1, modulates the expression of glucose sensors, including glucose transporter 2 (GLUT2) and glucokinase (GCK), in pancreatic β‐cells, which are critical for glucose‐induced insulin production [46]. These findings indicate that this pathway plays an important role in maintaining glucose homeostasis.

According to the study, it was found that an *P. strychnifolia* with a concentration of 200 mg/kg showed potential in combating diabetes. It achieved this by increasing the expression of the glucose transporter protein GLUT4 in the heart muscle, as well as GLUT2 and insulin in the pancreas of diabetic rats. The extract was administered continuously for 63 days without any adverse effects. However, it was noted that the medicine was not as effective as metformin in treating diabetes. Additionally, diabetic medicine can have negative adverse effects. Therefore, using 200 mg/kg of *P. strychnifolia* could be a good alternative treatment for diabetes. However, the future investigations should assess the molecular mechanisms underlying the hypoglycemic effect of *P. strychnifolia* extract through AMPK, Akt, and IRS‐1 signaling pathways.

## 5. Conclusions

Our study demonstrates the efficacy of *P. strychnifolia* in lowering blood glucose levels in diabetic rats. The critical compounds identified were 5,7‐trihydroxychromone‐3‐O‐α‐L‐rhamnopyranoside and 3,5,7,3′,5′‐pentahydroxy‐favanonol‐3‐O‐α‐L‐rhamnopyranoside, both of which exhibit inhibitory effects on α‐amylase and α‐glucosidase. At a 200 mg/kg dose, *P. strychnifolia* effectively reduced liver and kidney injury induced by STZ and increased insulin and GLUT2 and GLUT4 expressions. Moreover, the continuous administration of *P. strychnifolia* for 63 days did not result in mortality, clinical signs, changes in body weight, or alterations in organ weight. Therefore, *P. strychnifolia* may be an alternative to modulating type 2 diabetes without inducing adverse side effects.

## Author Contributions

Conceptualization, K.L‐O., U.L‐O., A.S., and K.B.; methodology, K.L‐O., U.L‐O., R.K., A.S., and K.B.; validation, K.L‐O. and K.B.; investigation, K.L‐O., U.L‐O., and A.S.; formal analysis, K.L‐O.; resource, K.L‐O. and K.B.; writing–original Draft, K.L‐O., U.L‐O., A.S., R.H., and K.B.; writing–review and editing, K.L‐O. and K.B.; supervision and funding acquisition, K.B.

## Funding

This work was supported by the Plant Genetics Conservation Project under the Royal Initiation of Her Royal Highness Princess Maha Chakri Sirindhorn, Walailak University grants (WUBG‐026/2565 and RSPG‐WU42/2566).

## Ethics Statement

*L. strychnifolia* (old name) was collected from Suan Ya Tai Tongnoppakhun herbal garden, which is owned by Mr. Srupsin Thongnoppakhun. The researcher, Dr. Kingkan Bunluepuech, has requested permission for collecting *L. strychnifolia* for research. The study was performed after receiving approval from the Walailak University Institutional Animal Care and Use Committee with Ref. No. WU‐ACUC‐65035, Walailak University.

## Conflicts of Interest

The authors declare no conflicts of interest.

## Data Availability

The data supporting the findings of this study are included within the article.
